# Protective Ventilation Improves Gas Exchange, Reduces Incidence of Atelectases, and Affects Metabolic Response in Major Pancreatoduodenal Surgery

**DOI:** 10.3389/fmed.2016.00066

**Published:** 2016-12-06

**Authors:** Vsevolod V. Kuzkov, Ludmila N. Rodionova, Yana Y. Ilyina, Aleksey A. Ushakov, Maria M. Sokolova, Eugenia V. Fot, Boris L. Duberman, Mikhail Y. Kirov

**Affiliations:** ^1^Department of Anesthesiology and Intensive Care, Northern State Medical University, Arkhangelsk, Russian Federation; ^2^Department of Anesthesiology, City Hospital # 1, Arkhangelsk, Russian Federation; ^3^Department of Surgery, Northern State Medical University, Arkhangelsk, Russian Federation

**Keywords:** protective ventilation, postoperative pulmonary complications, atelectasis, permissive hypercapnia, pancreatoduodenal surgery

## Abstract

**Background:**

Protective perioperative ventilation has been shown to improve outcomes and reduce the incidence of postoperative pulmonary complications. The goal of this study was to assess the effects of ventilation with low tidal volume (V_T_) either alone or in a combination with moderate permissive hypercapnia in major pancreatoduodenal interventions.

**Materials and methods:**

Sixty adult patients scheduled for elective pancreatoduodenal surgery with duration >2 h were enrolled into a prospective single-center study. All patients were randomized to three groups receiving high V_T_ [10 mL/kg of predicted body weight (PBW), the HVT group, *n* = 20], low V_T_ (6 mL/kg PBW, the LVT group, *n* = 20), and low V_T_ combined with a moderate hypercapnia and hypercapnic acidosis (6 mL/kg PBW, PaCO_2_ 45–60 mm Hg, the LVT + HC group, *n* = 20). Cardiopulmonary parameters and the incidence of complications were registered during surgery and postoperatively.

**Results and discussion:**

The values of V_T_ were 610 (563–712), 370 (321–400), and 340 (312–430) mL/kg for the HVT, the LVT, and the LVT + HC groups, respectively (*p* < 0.001). Compared to the HVT group, PaO_2_/FiO_2_ ratio was increased in the LVT group by 15%: 333 (301–381) vs. 382 (349–423) mm Hg at 24 h postoperatively (*p* < 0.05). The HVT group had significantly higher incidence of atelectases (*n* = 6), despite lower incidence of smoking compared with the LVT (*n* = 1) group (*p* = 0.017) and demonstrated longer length of hospital stay. The patients of the LVT + HC group had lower arterial lactate and bicarbonate excess values by the end of surgery.

**Conclusion:**

In major pancreatoduodenal interventions, preventively protective V_T_ improves postoperative oxygenation, reduces the incidence of atelectases, and shortens length of hospital stay. The combination of low V_T_ and permissive hypercapnia results in hypercapnic acidosis decreasing the lactate concentration but adding no additional benefits and warrants further investigations.

## Introduction

Postoperative pulmonary complications (PPC) can significantly worsen the outcomes of major surgery, thereby increasing the resource utilization and length of hospital stay (1). The benefits of the protective mechanical ventilation with low tidal volume (V_T_) resulting in improved outcome have been convincingly proved in patients with acute respiratory distress syndrome (ARDS) in large clinical studies and meta-analyses (2, 3). Respiratory support with protective V_T_ of 6–8 mL/kg to limit volumotrauma as well as setting of an adequate positive end-expiratory pressure (PEEP) to prevent atelectotrauma can be considered as key measures for both prevention and management of ARDS (4). Beyond the lower V_T_, the subgroup of protectively ventilated patients with ARDS with permissive hypercapnia might have certain additional benefits. The precise mechanism of this effect is not completely clear and may involve the suppression of inflammation, mitigation of cell apoptosis, and, finally, counteraction of the biotrauma (5–7).

During the past two decades, we observe a “paradigm shift” of preventive approach from tertiary, targeted on the prevention of the complication and mortality in ARDS, to secondary, aimed for the prevention of the development of PPC and ARDS *per se* (8). In patients with intact lungs, i.e., those without ARDS, the use of protective perioperative ventilation as “secondary” preventive measure can dramatically improve postoperative outcomes and reduce the risk of PPC (9). The prevention of PPC and its most severe form, postoperative ARDS, is of utmost interest in major abdominal surgery when patients have initially intact lungs but are in a risk group of postoperative respiratory adverse events (10, 11).

The important components of protective perioperative ventilation are low V_T_ and moderate PEEP targeting low plateau and driving pressures to avoid ventilator-associated lung injury (12). However, the independent contributing role of both the parameters as well as their interaction with specific pulmonary characteristics such as lung compliance and non-modifiable risk factors are to be further explored and discussed. In addition, the use of the relatively low V_T_ can be accidentally accompanied by permissive hypercapnia that can interact with systemic inflammatory response, biotrauma, and extrapulmonary organ function (6, 13).

The major pancreatoduodenal interventions include the extensive and complex resection of pancreas and duodenum involving hepatic and biliary structures. This branch of elective surgery may be potentially associated with a high risk of pulmonary and extrapulmonary postoperative complications due to history of smoking, alcohol consumption, high bleeding potential, hypoalbuminemia, and advanced age (14–16).

The goal of our study was to assess the effects of protective ventilation on hemodynamics, gas exchange, incidence of PPC and extrapulmonary complications, and clinical outcome. We hypothesized that the protective ventilation with low V_T_ results in the similar postoperative outcome and the incidence of PPC on Day 28 in the relatively homogenous population of the patients subjected to major pancreatoduodenal surgery. The secondary hypothesis was that protective ventilation combined with permissive hypercapnia does not improve organ functions compared with low V_T_ alone.

## Materials and Methods

The study protocol and informed consent were approved by the Ethical Committee of the Northern State Medical University, Arkhangelsk, Russian Federation. During the period of 2014–2016, 60 patients [28 females/32 males, age 54 (45–60) years] scheduled for major pancreatoduodenal surgery (mostly, extended pancreatic resection of pancreatic cancer or chronic calcific pancreatitis) with expected duration of the intervention exceeding 2 h were included into a prospective randomized study. All the patients were visited 12 h before the intervention in the surgical ward and signed an informed consent.

### Perioperative Ventilation

Before anesthesia and start of mechanical ventilation, the patients were randomized using the envelope method to three groups receiving either high V_T_ [10 mL/kg of predicted body weight (PBW); the HVT group, *n* = 20] or low V_T_ (6 mL/kg PBW; the LVT group, *n* = 20). An additional group combined low V_T_ with moderate permissive hypercapnia (V_T_ 6 mL/kg PBW and PaCO_2_ 45–60 mm Hg: the LVT + HC group, *n* = 20). In all the groups, PEEP of 4 cm H_2_O was set (Table 1).

**Table 1:** **Ventilator settings in the studied groups**.

Group	Acronym	Settings
High tidal volume	HVT	Tidal volume **10 mL/kg PBW** Initial respiratory rate was set at 12/min and tailored to achieve EtCO_2_ 35 mm Hg **Goal: PaO_2_ 90–150 mm Hg, PaCO_2_ 32–48 mm Hg**
Low tidal volume	LVT	Tidal volume **6 mL/kg PBW** Initial respiratory rate was set at 14/min and tailored to achieve EtCO_2_ 35 mm Hg **Goal: PaO_2_ 90–150 mm Hg; PaCO_2_ 32–48 mm Hg**
Low tidal volume + permissive hypercapnia	LVT + HC	Tidal volume **6 mL/kg PBW** Initial respiratory rate was set at 8–10/min and tailored to achieve EtCO_2_ above 45 mm Hg **Goal: PaO_2_ 90–150 mm Hg; PaCO_2_ 45–60 mm Hg**

*PBW, predicted body weight*.

The standard preoxygenation lasting for at least 3 min was performed in all the patients using 80% oxygen (Datex Ohmeda Avance, GE, Madison, WI, USA). Initial FiO_2_ was set at 30% to achieve SpO_2_ at least 95%. In case of SpO_2_ below 95%, FiO_2_ was increased with increment of 5% to achieve the target SpO_2_ value. In all the patients, the respiratory support was discontinued using standard criteria and spontaneous breathing trial. The tracheal extubation was performed in the ICU by an independent ICU physician. The criteria for discontinuation of respiratory support were as follows: the ability to tolerate 30 min of spontaneous breathing trial *via* the pressure support ventilation with pressure support level of 6–8 cm H_2_O, PaO_2_/FiO_2_ >200 mm Hg, spontaneous minute volume <10 L/min, and respiratory rate <30/min (f/V_T_ < 65 1/L and V_T_ > 6 mL/kg PBW) as well as normal body temperature, no obvious bleeding or anemia, hemodynamic stability, and adequate analgesia.

### Anesthesia

Before the interventions, all patients received premedication with sedative (phenazepam 1.0 mg) and antacid (omeprazol 20 mg). After transferring to the operating room, the catheterization of peripheral vein was performed and sedation with diazepam 5–10 mg intravenously was provided. The radial arterial line and thoracic epidural catheterization (Th_7_–Th_9_) were set in all patients. Epidural anesthesia (ropivacain 30–50 mg bolus with continuous infusion, fentanyl 100 μg bolus) was induced prior the start of surgery. General anesthesia was induced using propofol (1.5–2.0 mg/kg) and fentanyl (100 μg). Muscular blockade for the tracheal intubation was achieved with atracurium besilate (0.6 mg/kg). Thereafter, the anesthesia was maintained with sevoflurane 1.5–2.5 vol.% with fresh gas flow of 1 L/min and continuous infusion of fentanyl (100 μg/h) and atracurium (25 mg/h). Gastric tube and urinary catheter were set after the induction and intubation of the patients. Mean arterial pressure was maintained >55 mm Hg, if necessary using the titrated infusion of norepinephrine. Continuous infusion of a balanced crystalloid solution (4–5 mL/kg/h) was performed intraoperatively.

### Perioperative Measurements and Monitoring

Hemodynamics, gas exchange, and laboratory parameters were registered at the beginning of surgery, at the end of the intervention, and every 6–12 h during 72 h of the postoperative period. The invasive arterial blood pressure (radial artery), central venous pressure, and SpO_2_ were monitored continuously (B40 Patient monitor, GE Medical Systems, Freiburg, Germany). Inspiratory and end-expiratory sevoflurane concentration, FiO_2_ and FeO_2_, and EtCO_2_ were monitored using integrated monitor of anesthesia machine and Capnostream™ 20 monitor (Covidien, USA). Intra- and postoperatively, arterial and venous blood gases, lactate concentration, bicarbonate excess (BE), and hemoglobin concentration were registered.

Plain chest X-ray was performed as a standard procedure at 24 h of the postoperative period in the semi-recumbent position; the films were interpreted by an independent specialist. In cases when PPC (e.g., atelectasis, pleuritis, nosocomial pneumonia, etc.) were suspected, chest X-ray or computed tomography was performed within the period of observation up to Day 28 on request either in the ICU or in the radiology department.

The incidence of the postoperative complications including atelectases, postoperative ileus, nosocomial pneumonia, bleeding, and anastomosis leakage, as well as lengths of the ICU and hospital stay, and mortality were registered up to Day 28 after surgery.

### Statistics

The data distribution was assessed using Shapiro–Wilk test. The data are presented as median (25^th^–75^th^ percentiles). For data analysis, we used SPSS Statistics software (IBM, USA). Intergroup comparisons were performed using Kruskal–Wallis *H*-test followed by pair-wise *post hoc* Mann–Whitney *U*-test. The nominal data were compared using Pearson χ^2^-test followed with Exact Fisher’s test when appropriate. The intragroup differences were explored using Wilcoxon test. *p* values below 0.05 were regarded as statistically significant.

## Results

We did not find any significant baseline differences between the groups except for the history of smoking that was significantly lower in the HVT group (Table 2). The duration of both intra- and postoperative respiratory support as well as the overall duration of surgery were not different between all the three groups.

**Table 2:** **The characteristics of the patients**.

Data	HVT	LVT	LVT + HC	*p* values
Age, years	56 (48–61)	53 (45–63)	51 (41–58)	0.35
Weight, kg	70 (64–80)	62 (60–77)	69 (56–83)	0.54
Predicted body weight, kg	64 (56–71)	59 (53–67)	66 (52–71)	0.53
Gender (F/M)	8/12	11/9	9/11	0.63
Duration of surgery, min	160 (135–250)	190 (138–234)	225 (180–264)	0.27
Duration of mechanical ventilation, min	360 (270–525)	370 (265–499)	400 (295–473)	0.96
Length of ICU stay, h	44 (24–85)	43 (22–68)	45 (27–76)	0.71
Hospital stay, days	42 (25–51)	28 (21–38)^a^	31 (26–41)	**0.05**^a^
Smoking history, *n* (%)	6 (30)	14 (70)^b^	13 (65)	**0.03**^b^
All complications, *n* (%)	11 (55)	5 (25)	8 (40)	*0.13*
Atelectases, *n* (%)	6 (30)	1 (5)^c^	2 (10)	**0.04**^c^
Mortality, *n* (%)	1 (5)	0 (0)	2 (10)	0.32

*HVT, high tidal volume group; LVT, low tidal volume group; LVT + HC, high tidal volume group combined with hypercapnia. Data are presented as median (25^th^–75^th^ percentiles), numbers, or percentage*.

*p values are calculated using Kruskal–Wallis H-test with post hoc Mann–Whitney U-test when appropriate, or Pearson χ^2^-test followed with Exact Fisher’s test when appropriate for nominal data*.

*^a^p = 0.117 comparing all groups using Kruskal–Wallis H-test and p = 0.048 using Mann–Whitney U-test test between the HVT and LVT groups analyzed pair-wise*.

*^b^p = 0.025 comparing all groups using Pearson χ^2^-test and p = 0.026 using Exact Fisher’s test between the HVT and LVT groups*.

*^c^p = 0.047 comparing all groups using Pearson χ^2^-test and p = 0.038 using Exact Fisher’s test between the HVT and LVT groups*.

**Figure 1:**
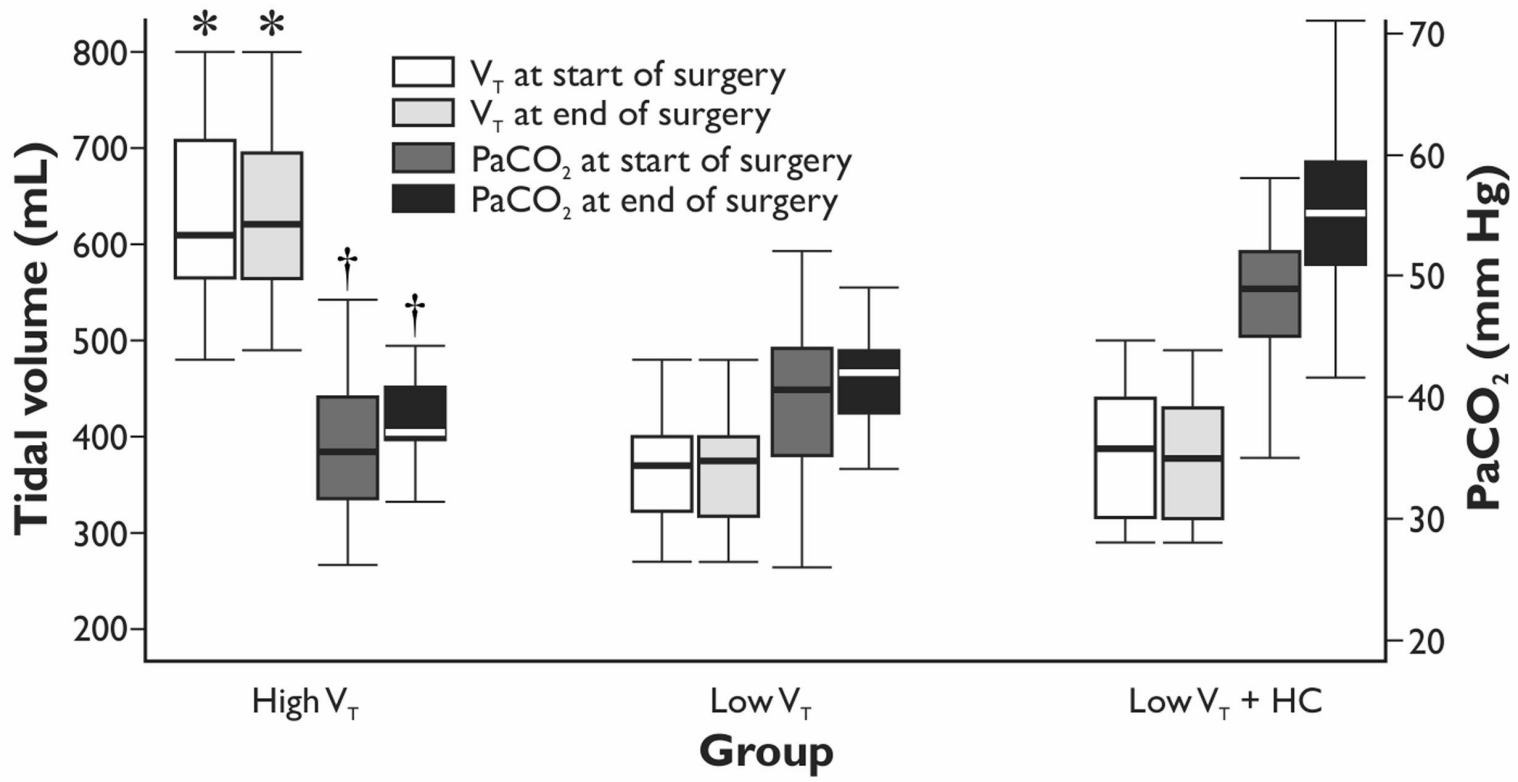
**The values of tidal volume and arterial partial pressure of CO_2_ in the groups at the beginning and end of the surgery**. Data are presented as median (25th–75th percentiles). *p* values are calculated using Kruskal–Wallis *H*-test followed by *post hoc* Mann–Whitney *U*-test. *p < 0.001 between the LVT, LVT + HC, and the HVT groups for the tidal volume set at the start and end of surgery. ^†^*p* < 0.001 between the LVT group and the LVT + HC group for PaCO_2_ at the start and end of surgery.

**Figure 2:**
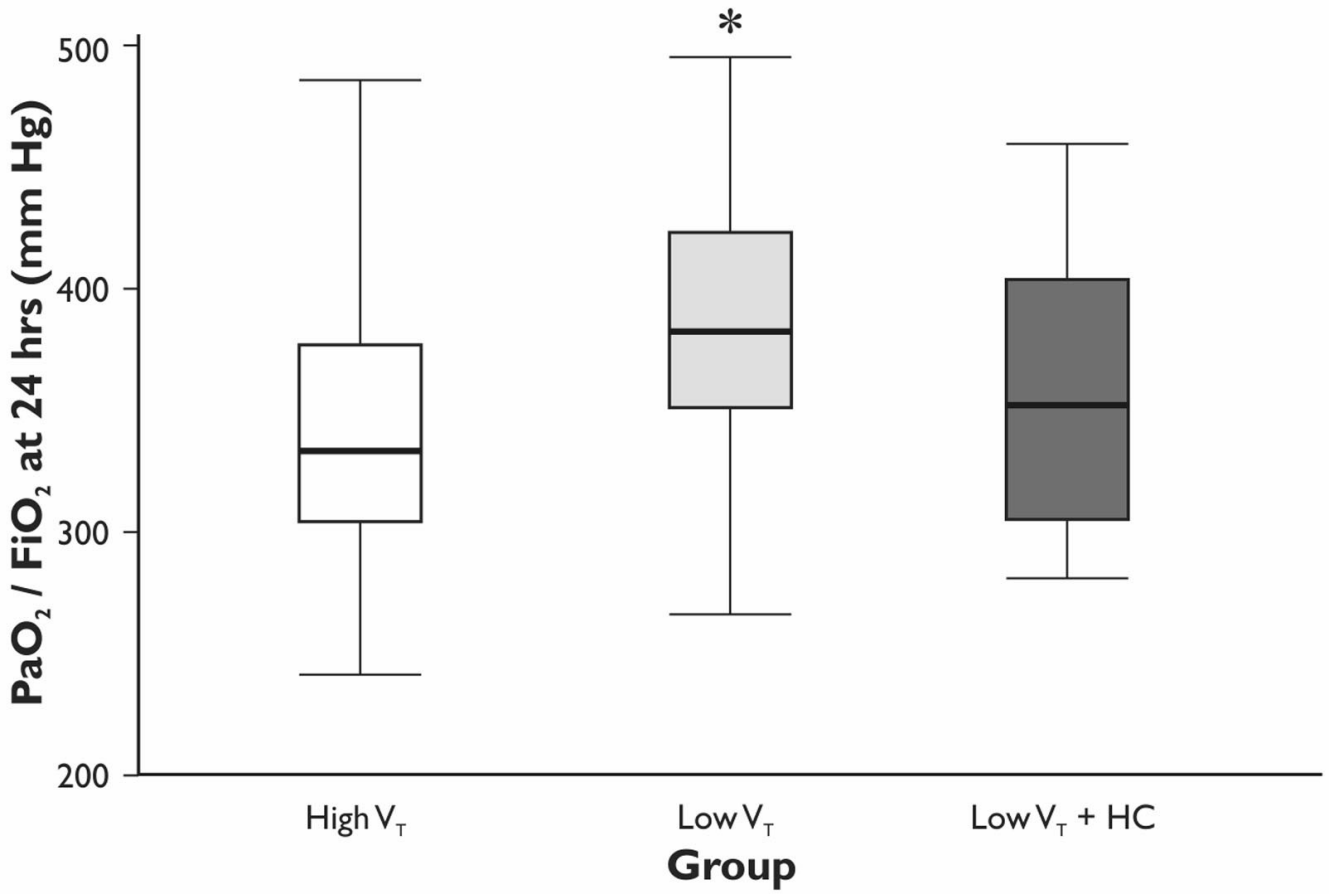
**The ratio of PaO_2_/FiO_2_ at 24 h after surgery**. Data are presented as median (25th–75th percentiles). *p* values are calculated using Mann–Whitney *U*-test between the HVT and the LVT groups; *p = 0.027 compared with the HVT group.

The values of V_T_ and PaCO_2_ at the start and completion of the surgery for the HVT, the LVT, and the LVT + HC groups are depicted in Figure 1. Compared with the HVT group, PaO_2_/FiO_2_ ratio at 24 h postoperatively was higher in the LVT group – 333 (301–381) vs. 382 (349–423) mm Hg (*p* = 0.027) but not in the LVT + HC group (Figure 2). Notably, the transient improvement of the postoperative oxygenation was achieved, regardless of the significantly higher incidence of smokers in the LVT group compared with the HVT group (*p* = 0.025; Table 2).

During surgery, we observed significantly increased V_E_ and peak pressures in the HVT group (Figure 1; Table 3). The LVT + HC group had significantly higher PaCO_2_ and EtCO_2_ compared with the other groups. In parallel with development of hypercapnia, arterial pH, BE, and lactate concentration reduced significantly (*p* < 0.03 and *p* < 0.02 compared with the HVT and the LVT groups, respectively, Table 3).

**Table 3:** **The characteristics of the patients**.

Parameter	Group	Stage					
		Start	End	6 h	24 h	48 h	72 h
Peak pressure, cm H_2_O	HVT	15 (14–19)	16 (15–19)	–			
	LVT	12 (11–13)*	13 (12–15)*				
	LVT + HC	12 (11–14)*	12 (11–15)*				
Driving pressure, cm H_2_O	HVT	5 (2–9)	4 (4–8)				
	LVT	5 (2–7)	5 (2–8)				
	LVT + HC	3 (2–6)	3 (2–8)				
Mean arterial pressure, mm Hg	HVT	65 (57–79)	78 (64–83)	–			
	LVT	73 (58–80)	74 (63–83)				
	LVT + HC	63 (56–81)	75 (68–78)				
Heart rate, 1/min	HVT	72 (67–83)	69 (63–79)				
	LVT	68 (57–76)	72 (61–83)				
	LVT + HC	65 (55–77)	72 (66–89)				
pH of arterial blood	HVT	7.41 (7.32–7.47)	7.30 (7.28–7.38)	7.36 (7.34–7.40)	7.42 (7.39–7.44)	7.43 (7.40–7.46)	7.44 (7.41–7.47)
	LVT	7.35 (7.30–7.40)	7.28 (7.25–7.32)	7.37 (7.35–7.39)	7.41 (7.36–7.45)	7.43 (7.41–7.46)	7.43 (7.40–7.44)
	LVT + HC	7.27 (7.23–7.33)*	7.16 (7.13–7.24)*	7.33 (7.31–7.37)^†^	7.40 (7.37–7.42)	7.42 (7.41–7.43)	7.44 (7.40–7.45)
BE, mmol/L	HVT	−2.2 (−3.9 to 0.0)	−5.9 (−7.5 to −3.1)	−5.2 (−7.8 to −3.1)	−2.2 (−4.0 to −0.6)	−1.3 (−3.7 to −0.2)	−2.6 (−4.8 to −0.4)
	LVT	−3.0 (−4.7 to −1.4)	−6.8 (−7.4 to −3.2)	−5.1 (−6.1 to −3.4)	−2.7 (−5.8 to −0.7)	−3.0 (−5.0 to −0.8)	−2.6 (−3.8 to −0.5)
	LVT + HC	−3.8 (−5.5 to −2.1)	−8.0 (−8.8 to −6.3)*	−6.1 (−7.8 to −5.4)^†^	−4.1 (−4.9 to −2.1)	−3.0 (−4.9 to −0.9)	−2.8 (−4.2 to −0.6)
Arterial lactate, mmol/L	HVT	0.8 (0.6–1.1)	1.1 (0.8–1.8)	1.7 (0.8–2.3)	1.2 (0.8–1.5)	0.8 (0.8–1.1)	0.8 (0.7–1.1)
	LVT	0.7 (0.5–0.8)	1.1 (0.7–1.5)	1.5 (1.0–3.1)	1.1 (0.9–1.5)	0.9 (0.7–1.2)	0.7 (0.6–1.2)
	LVT + HC	0.6 (0.5–0.7)*	0.7 (0.5–1.0)^*,†^	1.3 (1.0–2.4)	1.4 (1.0–2.2)	0.9 (0.7–1.6)	0.9 (0.7–1.2)
EtCO_2_, mm Hg	HVT	32 (28–35)	32 (29–35)	–			
	LVT	36 (35–42)	36 (34–38)				
	LVT + HC	45 (42–47)^*,†^	47 (45–50)^*,†^				

*HVT, high tidal volume group; LVT, low tidal volume group; LVT + HC, high tidal volume group combined with hypercapnia*.

*Data are presented as median (25th–75th percentiles), numbers or percentage. *p* values are calculated using Kruskal–Wallis *H*-test followed by *post hoc* Mann–Whitney *U*-test when appropriate*.

*p < 0.05 compared with the HVT group.

*^†^p < 0.05 compared with the LVT group*.

**Figure 3:**
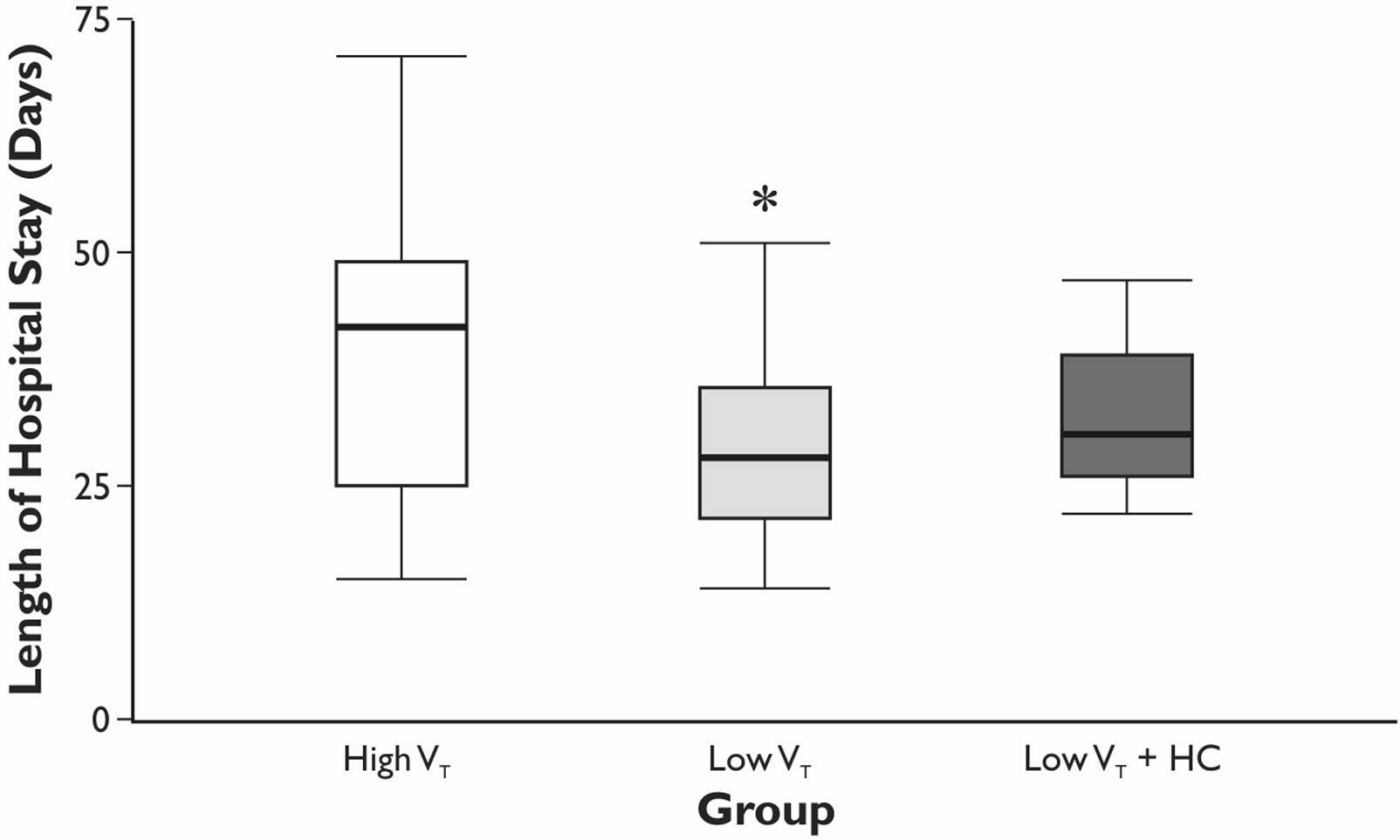
**The length of the hospital stay**. Data are presented as median (25th–75th percentiles). *p* values are calculated using Kruskal–Wallis *H*-test followed by *post hoc* Mann–Whitney *U*-test between the HVT and the LVT groups only; *p = 0.048 using Mann–Whitney *U*-test test between the HVT and LVT groups analyzed pair-wise.

**Figure 4:**
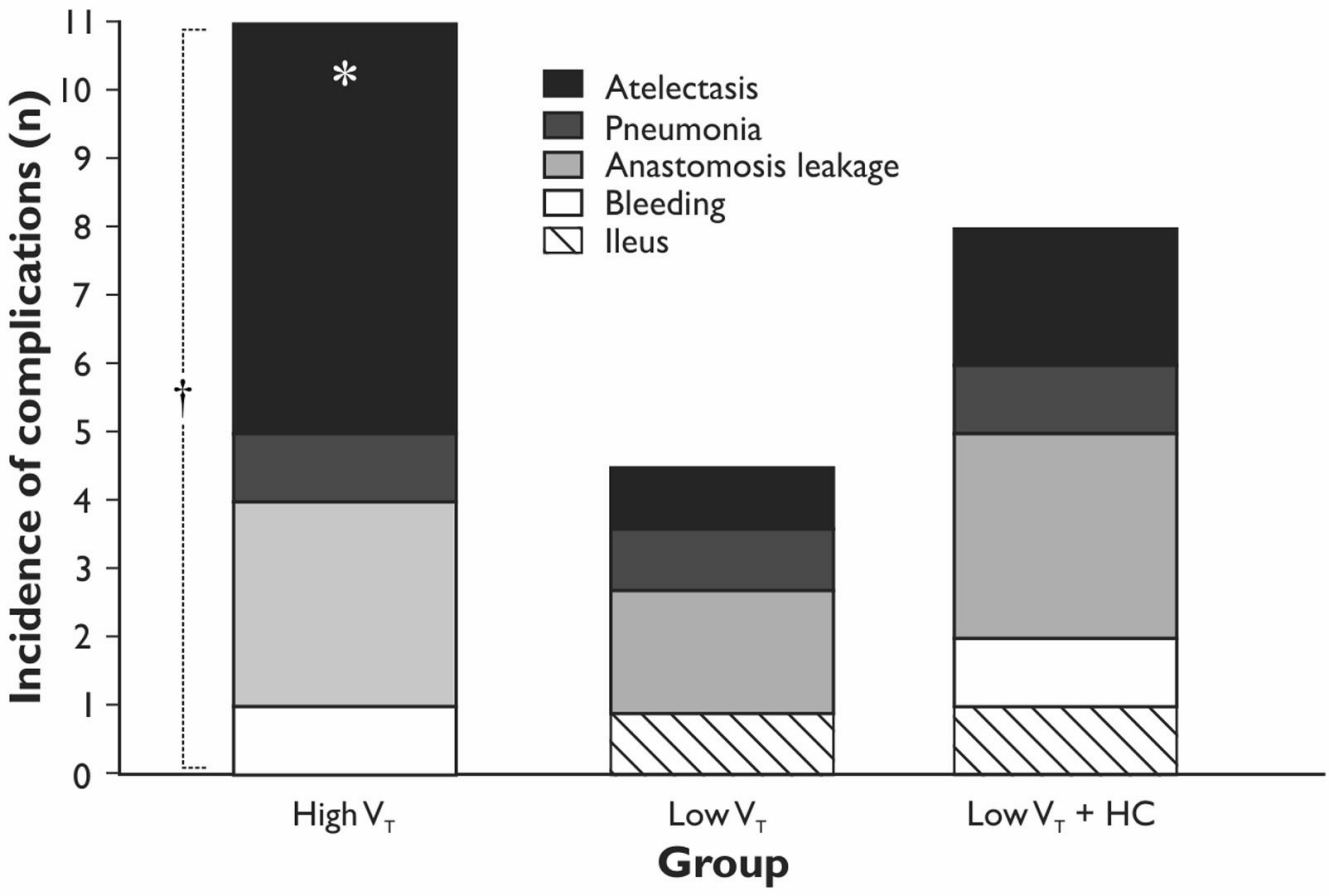
**The incidence of the postoperative complications in the study groups**. Data are presented as stacked numbers of the complications. *p* values are calculated using χ^2^-test between the HVT and the LVT groups; *p = 0.047 comparing all groups using Pearson χ^2^-test and *p* = 0.038 using Exact Fisher’s test between the HVT and LVT groups. ^†^*p* = 0.13 for incidence of all the complications between the HVT and the LVT groups.

The length of hospital, but not the length of ICU stay, was significantly longer in the HVT group compared with the LVT group when compared without LVT + HC group (Figure 3). The overall mortality at Day 28 was 5% (*n* = 1 in the HVT group and *n* = 2 in the LVT + HC group), and the overall incidence of postoperative complications was 40% (*n* = 24). We registered a tendency for higher incidence of the postoperative complications and significantly higher rate of the atelectases in the HVT group compared with the LVT group (Figure 4). We found no differences in the overall incidence of complications and atelectases between the HVT and the LVT + HC groups.

## Discussion

Our study demonstrated transiently improved postoperative oxygenation, reduced incidence of postoperative pulmonary atelectases, and shortened length of hospital stay in the patients ventilated with protective V_T_ of 6 mL/kg PBW during major pancreatoduodenal surgery. The combination of protective V_T_ with moderate hypercapnia and hypercapnic acidosis did not affect pulmonary function but could potentially interplay with perioperative acid–base balance.

The improvement of oxygenation (PaO_2_/FiO_2_) was relatively minor (15%) and transient as registered only at 24 h of the postoperative period in the LVT but not LVT + HC group. Moreover, we showed the decreased incidence of atelectases, mostly registered at 24 h postoperatively in parallel with changing PaO_2_/FiO_2_ ratio, and tendency to reduced overall incidence of the postoperative complications in the LVT group that was associated with increased length of the hospital stay in the group ventilated with high V_T_. In compliance with our results, Severgnini *et al*. have shown that open abdominal surgery lasting more than 2 h ventilation with relatively high V_T_ of 9 mL/kg and zero PEEP resulted in compromised pulmonary function, worsened oxygenation, and increased incidence of PPC in comparison with protective ventilation (V_T_ of 7 mL/kg of ideal body weight, PEEP of 10 cm H_2_O, and recruitment maneuvers) (10). In contrast, Treschan *et al*. demonstrated that the application of low V_T_ of 6 mL/kg PBW in major abdominal surgery did not improve postoperative lung function as compared with high V_T_ values of 12 mL/kg PBW with the similar PEEP level (5 cm H_2_O) (17). In the large randomized controlled trial, Futier et al. have demonstrated a reduction in the incidence of the major pulmonary and extrapulmonary complications within 7 days following major abdominal surgery by 17% in the protective ventilation (V_T_ 6–8 mL/kg PBW and PEEP 6–8 cm H_2_O) compared with the conventional ventilation group (V_T_ 10–12 mL/kg PBW and 0 PEEP) (11). In consistency with our results, this study convincingly proved that protective ventilation was associated with shorter length of hospital stay. The protective ventilation can prevent both volumotrauma, that triggers the PPC, and ARDS, as well as pulmonary cytokine release, i.e., biotrauma that can result in the propagation of systemic inflammatory response and distant organ injury. The involvement of the whole-body response to the perioperative pulmonary stress can explain the increased rate of extrapulmonary complications and, finally, the adverse clinical outcomes (11, 18). The potential overdistension of lungs in the HVT group could increase the risk of atelectases due to alveolar stretch and shear stress, thereby resulting in inflammation and repeated closure and opening of dependent areas (8, 18).

The ability of preventive low V_T_ to counteract the potential injurious and pro-inflammatory effects of inadvertent lung overdistension related to conventional ventilation is still a matter of debates (19, 20). Thus, Cai et al. showed by means of computed tomography that ventilation with protective V_T_ of 6 mL/kg alone without PEEP was not associated with any difference both in the incidence of atelectases and in oxygenation compared with the V_T_ of 10 mL/kg (21). In routine practice, low V_T_ is associated with the rather unjustified fair of atelectases, which probably could be counteracted by an adequate PEEP. Since our study included the patients with body mass index within the relatively normal range of 23.2 (21.3–28.4) kg/m^2^, the empiric and relatively low PEEP of 4 cm H_2_O could be considered adequate to reduce the risk of atelectases. As a result, low V_T_ was accompanied by a significant reduction of the atelectases incidence compared with the HVT group that makes this approach attractive for a wider use in clinical practice.

As noted, the PPC have been considered to be strongly associated with prolonged hospital stay (22), which was also confirmed by the presented results. Despite our study confirms the conclusions of several similar investigations showing that protective ventilation can improve gas exchange and lung mechanics and attenuate the risk of PPC and extrapulmonary adverse events (10, 11, 23), its results could contribute to the pool of the evidences favoring protective ventilation in major pancreatoduodenal surgery due to relatively homogenous patient population and insights into effects of permissive hypercapnia.

In our study, we induced a moderate degree of hypercapnia in the LVT + HC group aiming to prevent significant hemodynamic effects, risk of organ dysfunction, and increased consumption of anesthetic drugs. The patients assigned to this group did not show any additional improvement in oxygenation or reduced incidence of PPC and, namely, atelectases compared with both the LVT and the HVT groups. We found minor and transient metabolic effects in the LVT + HC group, namely, reduced arterial lactate concentration combined with respiratory acidosis and lower BE values. Hypercapnia and acidosis could interact with inflammation, modulate biotrauma, and attenuate ARDS that mostly explored so far in isolated lungs and *in vivo* experimental studies (7, 24, 25). In addition, the exact values of hypercapnic acidosis and hypercapnia are not settled ranging from 6.90 to 7.40 and 40 to 100 mm Hg, respectively, and the distinct mechanism of the protection remains unrevealed (26, 27). However, beyond the experimental attenuation of the cytokine release, hypercapnia can exert several deleterious effects *via* overproduction of nitric oxide, impaired plasma membrane repair, immunosuppression, and possible promotion of the bacterial growth (6). These effects combined with influence of hypercapnia on cardiovascular and central nervous systems can prevent physician to avoid this maneuver in patients without ARDS (28–30). The reduction in lactate concentration observed in our study can be explained by the metabolic acid–base effect of hypercapnic acidosis rather than any modification of organ perfusion. Indeed, it is suggested that the decreased lactate concentration during hypercapnia might actually result from the inhibition of phosphofructokinase activity, suppressed transport of lactic acid from muscles, and augmented rate of lactate oxidation (31–33). Therefore, the effects of hypercapnia associated with hypercapnic acidosis and low V_T_ on PPC incidence might worth further investigations to clarify the value and safety of this approach in the routine clinical practice.

### Limitations

The limitations of our study include the relatively small number of observations. Applying low V_T_, we did not consider the specific targets for pulmonary compliance, peak, plateau, and driving pressures that can also limit the applicability of the findings. The population of patients is relatively homogeneous in respect of the type of surgery but is heterogeneous for underlying pathology (both cancer- and not cancer-related interventions were included). Unfortunately, we could not explain clearly why the combination of low V_T_ and hypercapnia resulted in the potentially worse oxygenation, hospital length of stay, and the incidence of complications. We can hypothesize only that even moderate hypercapnia might be potentially detrimental for the patients without ARDS and multiple organ failure, and this risk forced us to limit the enrollment into this study after the intrinsic analysis.

## Conclusion

In major elective pancreatoduodenal surgery, preventive reduction of V_T_ to protective values results in transiently improved postoperative oxygenation, reduced incidence of atelectases, and shortened length of the hospital stay. The combination of low V_T_ and permissive hypercapnia leads to transient decrease in lactate concentration but does not add any substantial benefits to the outcome and organ function and warrants further investigations.

## Author Contributions

VK planned the design, performed data collection and analysis, and drafted the manuscript. LR planned the design, enrolled the patients, performed data collection and analysis, and drafted the manuscript. YI enrolled the patients, performed data collection, and drafted the manuscript. MS participated in the data collection and randomization of the patients. AU enrolled the patients, performed data collection, and drafted the manuscript. EF planned the design, performed analysis, and drafted the manuscript. BD participated in the data analysis and drafted the manuscript. MK planned the design of the study and drafted the manuscript.

## Conflict of Interest Statement

The authors declare that the research was conducted in the absence of any commercial or financial relationships that could be construed as a potential conflict of interest.
